# Adjusting the frequency of mammography screening on the basis of genetic risk: Attitudes among women in the UK

**DOI:** 10.1016/j.breast.2015.02.001

**Published:** 2015-06

**Authors:** Susanne F. Meisel, Nora Pashayan, Belinda Rahman, Lucy Side, Lindsay Fraser, Sue Gessler, Anne Lanceley, Jane Wardle

**Affiliations:** a Health Behaviour Research Centre, Dept. Epidemiology and Public Health, University College London, London, UK; b Dept. of Applied Health Research, University College London, London, UK; c Dept. of Women's Cancer, EGA UCL Institute for Women's Health, University College London, London, UK

**Keywords:** Breast cancer, Stratified screening, Population survey, Women's attitude

## Abstract

**Purpose:**

To explore public attitudes towards modifying frequency of mammography screening based on genetic risk.

**Methods:**

Home-based interviews were carried out with a population-based sample of 942 women aged 18–74 years in the UK. Demographic characteristics and perceived breast cancer (BC) risk were examined as predictors of support for risk-stratified BC screening and of the acceptability of raised or lowered screening frequency based on genetic risk, using multivariate logistic regression.

**Results:**

Over two-thirds of respondents (65.8%) supported the idea of varying screening frequency on the basis of genetic risk. The majority (85.4%) were willing to have more frequent breast screening if they were found to be at higher risk, but fewer (58.8%) were willing to have less frequent screening if at lower risk (t (956) = 15.6, p < 0.001). Ethnic minority status was associated with less acceptability of more frequent screening (OR = 0.40, 95% CI = 0.21–0.74), but there were no other significant demographic correlates. Higher perceived risk of BC was associated with greater acceptability of more frequent screening (OR = 1.71, 95%CI = 1.27–2.30).

**Conclusion:**

Women were positive about adjusting the frequency of mammography screening in line with personal genetic risk, but it will be important to develop effective communication materials to minimise resistance to reducing screening frequency for those at lower genetic risk.

## Introduction

Breast cancer screening by mammography has been identified as reducing deaths from breast cancer; nonetheless, false positives, overdiagnosis and overtreatment have all been identified as potential harms [1,2]. Routine screening is usually recommended for women above age 50 [http://www.cancerscreening.nhs.uk/; http://www.who.int/cancer/detection/variouscancer/en/; http://www.cdc.gov/cancer/dcpc/prevention/screening.htm]; although women with a strong family history of breast cancer may be offered screening from an earlier age [3] http://www.cancerscreening.nhs.uk/; http://www.cdc.gov/cancer/dcpc/prevention/screening.htm). However, approximately 20% of all breast cancers occur in women younger than age 50 [4]; with a substantial proportion in women without a family history. These cancers tend to be aggressive with a poorer prognosis [5,6], and therefore early identification, irrespective of known family history, might be beneficial.

Given the number of genetic markers for breast cancer risk that have been identified [7–9], incorporating genetic risk assessment into current mammography screening has been proposed as one way to maximise benefits and minimise harms [10]. Modifying screening eligibility and frequency to account for genetic risk could make it possible to ‘tailor’ screening and risk management efforts to those at the highest risk, for example by shortening screening intervals, or by offering screening using alternative modalities such as Magnetic Resonance Imaging (MRI) and ultrasound. At the same time, it would minimise exposure to potential harms of screening for those at the lowest risk; for example by starting screening later or by making a recommendation against routine mammography screening in this group. Risk-stratified mammography screening based on genetic risk could therefore be superior to current age-stratified approaches [11,12].

However, implementation of genomic risk-stratified breast screening would require the support of the wider public. The public is generally very enthusiastic about screening [13,14]. Women perceive high benefits of mammography screening [15,16]; reflected in the high attendance rates (around 70%) across countries [17–19]; although lower socioeconomic status (SES) and ethnic minority status have both been associated with lower participation rates [20–22]. Perceived risk of breast cancer has been cited as encouraging some individuals to be screened, while deterring others [16,23,24]; so predicting the impact of giving genetic risk information on screening uptake is difficult. There has also been attention to public perceptions of a ‘right to be screened’, which may militate against the acceptability of reducing breast screening frequency for those at the lowest risk.

Few studies to date have investigated public attitudes to ‘personalised’ cancer screening, despite calls for empirical research on the topic [10,25]. One qualitative study in the Netherlands found that women were supportive of both increases and reductions in breast screening frequency, although there was an important proviso that any woman who was worried about breast cancer despite having low genetic risk, should still be able to access screening [26]. A small qualitative study in the UK found enthusiasm for risk-stratified ovarian cancer screening based on genetic risk [27], but these findings cannot be assumed to generalise to an existing and very popular mammography breast screening programme; especially given evidence from two studies that show that information on overdiagnosis is not a deterrent for mammography screening for most women [14,28].

Given that research is under way to test the feasibility of ‘personalising’ mammography screening based on individual risk factors, including genetic risk [29], the primary aim of this study was to investigate public attitudes towards amending the frequency of breast cancer screening based on genetic risk in the current UK National Health Service Breast Cancer Screening Programme. Data were from a large, population-based study, making it possible to identify demographic and personal predictors of support for raising or lowering frequency of cancer screening based on genetic risk.

## Methods and procedure

### Sample

Data were collected by adding a question module on ‘genetics and screening’ to the ‘Opinions and Lifestyle’ survey, which is conducted monthly by the UK Office of National Statistics (ONS) on behalf of government departments, non-governmental agencies and academic institutions. Each month, 2010 households are identified from the Royal Mail's Postcode Address File using stratified random probability sampling. Selected addresses are contacted up to eight times at different times and days of the week to maximize response rates. One person aged over 16 from each household is randomly chosen to complete a computer-assisted face-to-face interview with trained researchers. Questions on the ‘genetics and screening’ module were included in two data collection waves, January and March 2014. Although questions were read out, we used the Flesch Reading Ease formula and the Flesch-Kincaid Grade Level formula to estimate comprehension. This produced a score of 68; conferring a reading level expected in grade 6 (age 12).

### Measures

The module was introduced with a short explanation about genes and risk-stratified cancer screening that had been agreed with ONS: ‘*Genes contain the ‘instruction manual’ of life, called DNA. Genes are passed from parents to their children.* Nowadays, it is possible to predict whether someone is likely to develop certain diseases by looking at their genes. This is called genetic testing’. No information was given on current breast cancer screening approaches or potential harms of screening.

#### Outcome variables

One question addressed attitudes to risk-stratified breast cancer screening: *‘It is possible that breast screening frequency could be varied depending on whether a woman is at higher or lower genetic risk of breast cancer. What do you think of the idea of varying the frequency of breast screening’ (‘very bad idea’; ‘bad idea’; ‘not sure’; ‘good idea’; ‘very good idea’)*. Responses were dichotomized into very bad idea/bad idea/not sure vs. good idea/very good idea to reflect not supporting vs. supporting personalized risk-stratified breast cancer screening.

Personal acceptability of modified breast screening frequency was assessed with two questions: *‘Would you personally be happy to have your breast screening more often if you were found to be at higher genetic risk of breast cancer’, and ‘Would you personally be happy to have your breast screening less often if you were found to be at lower genetic risk of breast cancer’ (‘very unhappy’; unhappy’; ‘not sure’; ‘happy’ and ‘very happy’)*. For some analyses, very unhappy/unhappy/not sure were combined for comparison with happy/very happy to reflect low and high acceptability of varying the frequency of breast cancer screening.

Attitudes towards genetic testing generally were assessed to be sure that acceptability of stratified screening was not influenced by views about genetic testing, using the question: ‘*B*ased on what you know, do you think genetic testing will do more good than harm, or more harm than good? Response options were ‘Do more good than harm’, ‘do more harm than good’, ‘not sure/it depends’ and ‘I have never heard of genetic testing’. Responses were coded as *‘Do more good than harm’* vs. *‘do more harm than good/it depends’*. Participants who responded that they had never heard of genetic testing were excluded from further analyses (n = 14). This question was taken from previous surveys that investigated public attitudes to genetic testing [30,31].

#### Predictor variables

Perceived relative risk of breast cancer was assessed with one question: ‘*Compared with other women of your age, what do you think are your chances of getting breast cancer*’ with response options of: ‘*much lower than others*’, *‘lower than others’, ‘the same as others’, ‘higher than others’*, *‘much higher than others’*. For the current analyses, we treated this variable as continuous, so as to not lose power.

Demographic data were provided by ONS from their standard survey items (http://www.ons.gov.uk/ons/%20ons/index.html). Age was coded as ≤50 vs. >50 years; women >50 years could have already been invited for breast screening, as part of the national breast screening programme in the UK. Ethnicity was classified as ‘White’ vs. ‘ethnic minority’ because the individual ethnic minority sub-groups were small. Educational attainment was classified as university degree or equivalent vs. below university degree level. Marital status was coded as married/cohabiting vs. single/widowed/divorced. Unfortunately, we had no information on past uptake of screening; or family history of breast cancer.

### Statistical analyses

We included women aged 18–74 to reflect the population who could be eligible for risk-stratified breast cancer screening. Statistical analyses were carried out using the Statistical Package for Social Sciences (SPSS) version 20.0 (SPPS Inc., Chicago, IL). We explored demographic and personal predictors for each outcome variable in univariate χ^2^ analyses. Multivariate logistic regression was used to establish the associations of all described demographic characteristics and beliefs about genetic testing; as well as attitudes to risk-stratified mammography screening. Analyses were repeated including perceived risk. Bonferroni corrections were employed to correct for multiple testing and α was set at 0.05/5 = 0.01.

## Results

In the January wave, 8% (n = 166) of the 2010 selected households were not eligible because they were businesses or empty properties. Of 1844 eligible households, 9% (n = 171) could not be contacted and 33% (n = 608) declined to take part in the ONS survey. In the March wave, 1853 households were eligible (92%). Of those, 13% (n = 237) could not be contacted and 31% (n = 578) chose not to take part. Therefore, the overall response rate was 57%; comparable to previous ONS surveys (http://www.ons.gov.uk/ons/index.html). The final female sample was n = 1095.

After excluding individuals ineligible by age (aged below 18 years and above 74 years; n = 138), or missing data (n = 15) the final sample for analysis consisted of 942 women aged 18–74 (mean 47 years; SD = 15.6). Most were ‘White’ (92.8%, n = 874), over half were married or cohabiting (54.1%, n = 510), and over a quarter (26.8%, n = 252) were at least university educated. In common with other survey studies [32], education was higher in the sample than in the general population; other demographic characteristics were comparable to the UK population of women aged 18–74 (ONS Census 2011: (http://www.ons.gov.uk/ons/guide-method/census/2011/index).

Most women (67.7%) held the belief that ‘genetic testing causes more good than harm’, 7.4% believed that it ‘causes more harm than good’, and 23.5% thought that ‘it depends’. The belief that ‘genetic testing causes more harm than good’ was unrelated to any demographic factors or to perceived risk (data not shown).

### Attitudes to risk-stratified breast cancer screening

Over two thirds of respondents (65.8%) thought that varying the frequency of breast cancer screening to take into account personal genetic risk was a ‘good’ or ‘very good’ idea. Other respondents rated it as ‘bad (13.4%), ‘very bad’ (4.0%), or were ‘not sure’ (16.8%).

The majority (85.4%) would be willing to have more frequent breast screening if they were found to be at higher risk. Significantly fewer women (58.8%) would be willing to have less frequent breast screening if they were found to be at lower risk (t (956) = 15.6, p < 0.001) (See Table 1).

Women with university education were more supportive of varying breast cancer screening frequency in univariate analyses (p = 0.023), but the association became non-significant after adjustment for covariates and multiple testing (p = 0.121) (see Table 2). Ethnic minority status was associated with less positive attitudes towards more frequent screening at a higher genetic risk in univariate and multivariate analyses.

### The impact of perceived risk on attitudes to risk-stratified breast cancer screening

Of 858 women who answered the question on perceived breast cancer risk, 16.5% (n = 135) felt at ‘much lower’ or ‘lower risk’ of breast cancer, most felt that their risk was ‘the same’ (73.5%, n = 631), and very few women felt that they were at ‘higher’ or ‘much higher’ risk of breast cancer (9.9%, = 85) than other women of their age group (mean score: 2.91, SD = 0.6). Higher perceived risk of breast cancer was associated with more positive attitudes towards more frequent breast cancer screening in the multivariate analysis, but had no impact on attitudes towards varying screening frequency in general, or on reducing screening frequency for those at lower risk (Table 2).

## Discussion

This is the first population-based study investigating women's attitudes to risk-stratified mammography screening based on genetic risk assessment. Although the majority of women were enthusiastic about a risk-stratified approach to breast screening, this was particularly with respect to increasing screening frequency for those at higher genetic risk. Fewer were supportive of a reduction in screening frequency for those at lower risk.

Since most women were of the opinion that genetic testing would ‘cause more good than harm’, it is unlikely that the different reactions to more vs. less frequent screening are related to the use of genetic testing *per se*, and more likely they are due to the perceived benefits of mammography screening. One possibility is that it reflects a notion of an ‘acquired right’ to screening, as described by Henneman and colleagues [26]. Studies of attitudes to overdiagnosis [28,33] suggest that many women take a ‘better safe than sorry’ approach to cancer screening; making them more accepting of false-positive results and over-diagnosis. Further research is needed to explore the reasons behind this finding and discover how to best communicate any changes to breast screening recommendations.

Ethnic minority status was associated with negative attitudes towards attending screening more frequently with a higher genetic risk, although there was no relationship with attitudes towards risk-stratified screening in general, or towards reducing screening frequency for those at lower risk. This suggests that the prospect of more frequent mammography screening may be problematic for some subgroups. Breast screening attendance has historically been lower for women from ethnic minority groups [34], with notions of privacy and modesty found to be barriers to breast screening participation [35,36]. Furthermore, cancer fatalism (i.e. the belief that cancer is inevitable) is higher in some ethnic groups, which has also been suggested as a reason to forego breast cancer screening [37,38]. However, the small numbers of ethnic minority women in the present sample – approximately reflecting population levels – made it impossible to compare individual ethnic groups. This could be explored in future research.

Although perceived risk was associated with more positive attitudes to more frequent breast-screening, it was not associated with any other variables. This replicates findings from earlier studies, including those with high risk groups [23,24,39], and is in line with protection motivation theories of health behaviour which outline high perceived risk of a disease as one important motivator for initiation of risk-reducing behaviours [40].

This study had some strengths. The questions were included in a large, monthly survey which is broadly representative of the population of women aged 18–74. Researcher or participant bias was unlikely since it had been conducted by an external agency (ONS), and consent was based on the whole survey and not a specific module; although participants could withdraw at any time. We adjusted analyses for multiple testing which gives confidence in the current findings. It also had important limitations. Because of survey constraints, women were given little information on how the risk-stratified approach would work in practice, and no information on current breast screening approaches (screening modality, frequency, potential harms of breast cancer screening). Knowledge of mammography screening may vary across the population and may affect outcomes, but this was not assessed. Furthermore, given that some women hold very strong beliefs about breast screening, this information may not have been sufficient to elicit more in-depth considerations about benefits and harms of a risk-stratified approach. Although we used established measures to assess readability of the questions, all scenarios were hypothetical which may have been difficult to understand for some women; particularly if English was not their first language. Thirdly, no open questions were used, so answers could not be explored in greater detail. Lastly, we did not assess current screening behaviour and family history of breast cancer which may have affected outcomes. For example, women who reported a higher perceived risk of breast cancer may have had a family history which would have justified their perceptions. Future research could explore this in more detail. However, despite the limitations, these findings give a first indication of general attitudes towards amending a much valued breast-screening programme.

## Conclusion

The results of this study indicate that women are generally positive towards risk-stratified mammography screening based on genetic risk assessment; although it will be important to develop effective communication materials to minimise resistance to reduction in screening frequency for those at lower genetic risk. The findings give confidence that the general public would support the integration of novel genomic technologies into mainstream healthcare.

## Conflict of interest statement

The authors have no conflict of interest to declare.

## Figures and Tables

**Table 1:** Univariate analyses (χ^2^-test) of support of risk-stratified breast cancer screening.

Variable	Good/very good idea (vs. bad/very bad idea/not sure) to vary frequency of BC screening by risk		Happy/very happy (vs. very unhappy/unhappy/not sure) to have more frequent BC screening if at higher risk		Happy/very happy (vs. very unhappy/unhappy/not sure) to have less frequent BC screening if at lower risk	
	% (n)	p-value	% (n)	p-value	% (n)	p-value
Total (n = 942)	65.8 (619)		85.4 (804)		58.8 (554)	
Age						
>50	67.6 (354)	0.125	84.4 (352)	0.395	56.2 (235)	0.225
≤50	63.2 (264)		86.1 (452)		60.9 (319)	
Ethnicity						
White	65.7 (576)	0.862	86.3 (757)	0.002	58.7 (515)	0.840
Ethnic minority	64.6 (42)		72.3 (47)		60.0 (39)	
Education						
University degree	71.4 (180)	0.023	87.4 (222)	0.279	63.6 (161)	0.068
Below university degree	63.5 (438)		84.6 (582)		57.0 (393)	
Marital status						
Married/cohabiting	64.6 (329)	0.497	85.5 (435)	0.917	59.1 (300)	0.869
Single/widowed/divorced	66.7 (289)		85.2 (369)		58.5 (254)	
Perceived risk of breast cancer(n = 858)						
Much lower than others	76.9 (21)	0.297	63.0 (19)	0.002	66.7 (19)	0.875
Lower than others	73.7 (85)		79.8 (91)		62.3 (71)	
The same as others	65.1 (410)		86.0 (542)		59.7 (377)	
Higher than others	69.3 (52)		90.7 (68)		58.7 (44)	
Much higher than others	60.0 (6)		100.0 (10)		70.0 (7)	

**Notes:** Abbreviations: BC = Breast cancer.

**Table 2:** Multivariate logistic regression of predictors of attitudes to risk-stratified breast cancer screening including genetic risk.

Variable	Good/very good idea (vs. bad/very bad idea/not sure) of varying frequency of BC screening by personal risk			Happy/very happy (vs. very unhappy/unhappy/not sure) to have BC screening more often if found at higher risk			Happy/very happy (vs. very unhappy/unhappy/not sure) to have BC screening less often if found to be at lower risk		
	OR	95% CI	p-value	OR	95% CI	p-value	OR	95% CI	p-value
Age									
>50	1			1			1		
≤50	1.17	0.88–1.58	0.280	1.25	0.84–1.85	0.273	1.18	0.89–1.57	0.228
Ethnicity									
White	1			1			1		
Ethnic minority	0.86	0.49–1.50	0.598	0.40	0.21–0.74	0.003	1.03	0.59–1.78	0.911
Education									
University degree	1			1			1		
Below university degree	0.768	0.55–1.07	0.121	0.82	0.52–1.28	0.378	0.82	0.60–1.14	0.245
Marital status									
Married/cohabiting	1			1			1		
Single/widowed/divorced	1.28	0.96–1.71	0.096	1.01	0.69–1.49	0.954	1.02	0.72–1.26	0.879
Perceived risk of breast cancer^b^	0.83	0.66–1.05	0.135	1.71^a^	1.27–2.30	<0.001	0.94	0.75–1.18	0.592

Abbreviations: BC = Breast cancer, OR = odds ratio, CI = 95% confidence interval. Notes: Results are mutually adjusted for age, ethnicity, education, marital status, and perceived risk of breast cancer.

a Significant after Bonferroni corrections.

b OR refers to trend for this variable.
